# Randomized Trial: A Pilot Study Investigating the Effects of Transcendental Meditation and Yoga Through Retinal Microcirculation in Cardiac Rehabilitation

**DOI:** 10.3390/jcm14010232

**Published:** 2025-01-03

**Authors:** Adam Saloň, Karin Schmid-Zalaudek, Bianca Steuber, Maximilian Elliot Rudlof, Till Olaf Bartel, Petra Mächler, Andreas Dorr, Rainer Picha, Per Morten Fredriksen, Benedicta Ngwenchi Nkeh-Chungag, Nandu Goswami

**Affiliations:** 1 Gravitational Physiology and Medicine Research Unit, Division of Physiology & Pathophysiology, Medical University of Graz, 8010 Graz, Austria; 2 Faculty of Applied Ecology, Agricultural Sciences and Biotechnology, Inland Norway University of Applied Sciences, 2624 Lillehammer, Norway; 3 Vascular Biology Center, Augusta University, Augusta, GA 30912, USA; 4 Rehabilitation Center for Cardiovascular Disease, 8061 St. Radegund, Austria; 5 Department of Biological and Environmental Sciences, Faculty of Health Sciences, Walter Sisulu University PBX1, Mthatha 5117, South Africa; 6 Center for Space and Aviation Health, College of Medicine, Mohammed Bin Rashid University of Medicine and Health Sciences, Dubai 505055, United Arab Emirates

**Keywords:** cardiac rehabilitation, exercise therapy, meditation, yoga, retinal imaging

## Abstract

**Background/Objectives:** Cardiovascular diseases are a leading cause of death, and psychosocial stress is considered a contributing factor to these issues. With the rising number of heart surgeries, proper rehabilitation post-surgery is essential. Previous studies have demonstrated the positive impact of yoga and transcendental meditation on the cardiovascular system. This pilot study aimed to investigate the effects of yoga and transcendental meditation on retinal microcirculation in cardiac patients before (admission), after (discharge), and following (3 weeks after discharge) rehabilitation. **Methods**: This study examined changes in retinal microcirculation in three rehabilitation groups of patients after heart surgery. The control group received standard exercise therapy, while the meditation group incorporated 20 min of meditation, and the yoga group incorporated 20 min of yoga practice, twice per day for the duration of four weeks of rehabilitation. Retinal images were captured using a non-mydriatic digital retinal camera (Canon CR-2, Canon Medical Systems Europe B.V., Netherlands), and the microcirculation parameters central retinal artery equivalent, central retinal vein equivalent, and artery-to-vein ratio were analyzed using MONA REVA software ((version 2.1.1), VITO, Mol, Belgium). Repeated measures ANOVA was performed to evaluate differences between the three groups in the course of rehabilitation. **Results**: None of the parameters revealed significant differences in retinal microcirculation between the three rehabilitation groups. **Conclusions**: The study evaluating changes in retinal microcirculation, as an indicator of central circulation in cardiac patients undergoing rehabilitation, did not observe any significant changes. As yoga and meditation are underestimated approaches in cardiac rehabilitation, this pilot study acts as a basis for providing preliminary information for future studies to encourage the research community to fill the gap in this area.

## 1. Introduction

The review by Roth and colleagues, mapping the burden of cardiovascular diseases from the years 1990 to 2019, showed a continued elevation of cardiovascular mortality [1]. On 1 June 2022, the National Heart, Lung, and Blood Institute reported that each year, more than 2 million people worldwide undergo open-heart surgery [2]. The global improvement in healthcare is contributing to a growing population of elderly individuals, suggesting that these numbers may continue to rise in the future. Regarding complete recovery after heart surgery, an important part is played by a controlled rehabilitation program. There are different approaches to rehabilitation after heart surgery. They usually include exercising, emotional/psychological support, and lifestyle education to reduce the heart disease risk [3,4]. However, psychosocial stress and stress conditions are also associated with worsening cardiovascular health and should be prevented [5,6,7].

According to the International Yoga Federation, about 300 million people in the world practice yoga [8]. Among the major benefits of yoga are the enhancement of strength and flexibility, the improvement of respiratory and cardiovascular function, a reduced level of stress, and the improvement of sleeping and quality of life [9,10,11,12]. The National Institute of Health’s national survey found that over 55% of yoga users report better sleep and that yoga reduces stress in over 85% of users [13].

Moreover, the estimate of people practicing meditation globally is between 200 and 500 million [14]. Data acquired between 2012 and 2017 show that the popularity of meditation is growing rapidly and has equaled the popularity of yoga, and it is highly likely that today it is already more popular than yoga itself [15]. It is a broadly known fact that even though yoga and meditation are related practices, they are not the same. While yoga involves physical postures, and breathing exercises, and can often include different meditation techniques, meditation itself focuses on mental concentration and mindfulness without physical movement and is practiced on its own, independently of yoga. The most common beneficial effects of meditation include improvement in negative mood and depression, a reduction in fatigue, the amelioration of blood pressure (BP) and lipid profiles, and a decrease in insulin resistance [16,17,18].

Therefore, the positive effects of meditation and yoga on mental and cardiovascular health are known [9,10,11,12,19,20,21]. While general meditation encompasses a variety of practices without a single prescribed method, transcendental meditation (TM) uses a specific mantra and follows a standardized technique. Previous research showed that yoga, as well as meditation, are effective approaches to cardiovascular health improvement, mostly decreasing heart rate (HR) and performing BP regulation. Other studies also confirmed the favorable effects of these practices on HR variability (HRV), anthropometric parameters, the lipid profile, and insulin resistance [18,22,23,24]. Several independent studies suggested that the mechanism responsible for this action is most probably hidden behind induced parasympathetic or reduced sympathetic activity after yoga or meditation [12,24,25,26,27].

The evaluation of retinal microvessel parameters is an innovative approach that enables the non-invasive and quick assessment cardiovascular health. This technique looks on the cardiovascular system through the parameters of small, repulsive arterioles and venules in the retina. The change in these parameters reflects modifications in retinal vessels. Previous research confirmed that these changes can reflect and predict future changes in the bigger vessels of the cardiovascular system [28,29,30,31,32,33,34,35,36,37]. Therefore, this technique is a promising approach with which to speed up the diagnosis and treatment of cardiovascular diseases. Data collection, as well as the verification of this methodology, is a big necessity in order to implement this approach to general practice.

**Figure 1:**
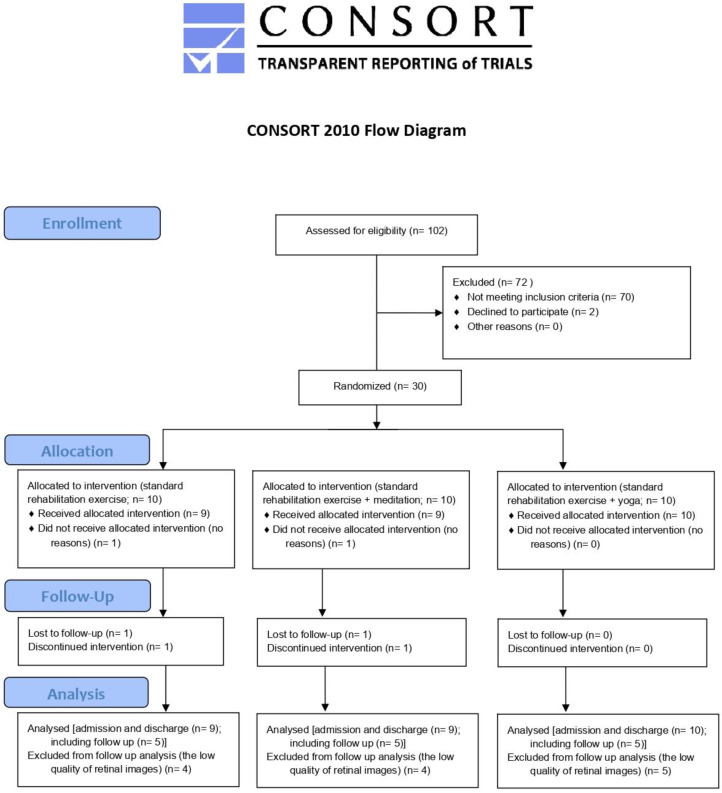
CONSORT flow diagram.

Significant epidemiological and mechanistic investigations have shown that psychosocial stress plays a role in the development and clinical occurrence of cardiovascular disease (CVD) [38]. The alleviation of psychosocial stress is considered one of the primary objectives in cardiac rehabilitation, alongside the promotion of physical activity and the enhancement of dietary habits [39]. Meditation and yoga represent two distinct approaches that can be employed to enhance mindfulness and alleviate stress and their implementation during post-heart surgery rehabilitation could improve the quality of the rehabilitation process and, in the end, the health of the patients. Although yoga and meditation are practiced by millions of people around the world and their positive effects have been recorded, the use of these approaches remains underestimated by the research community. As mentioned by Thabane and colleagues, the common idea behind how a pilot study is presented by multiple sources is the conduction of a study in advance of a larger, more comprehensive investigation [40]. They also suggested that feasibility should be a part of the pilot study. In accordance with this, the present pilot study was conducted in a way that meets CONSORT (Consolidated Standards of Reporting Trials) criteria ([41], Figure 1, Supplement Table S1). The objectives of this study were as follows: the investigation of the changes in retinal microvasculature in cardiac patients undergoing cardiac rehabilitation; the comparison of three different rehabilitation approaches (standard rehabilitation therapy, standard rehabilitation therapy plus meditation, and standard rehabilitation therapy plus yoga) in cardiac patients; and the examination of the possibility of capturing changes caused by cardiac rehabilitation using retinal imaging, which represents a novel and promising strategy in cardiovascular health evaluation [42,43,44,45,46,47]. We also aimed to provide preliminary data about the given topic and highlight the importance of this research gap to encourage further research.

This study hypothesizes that changes in retinal microvasculature will be observed in cardiac patients undergoing cardiac rehabilitation; there will be differences in outcomes among the three rehabilitation approaches; retinal imaging will be a reliable method for capturing changes over the study and evaluating cardiovascular health in a non-invasive manner; and this study will provide preliminary data with in order to address the importance of this research and contribute to future research endeavors.

## 2. Methods

This pilot, randomized, and single-blinded (investigator) clinical study was carried out from October to December 2019. Patients who had experienced coronary events were eligible for in-patient rehabilitation at the Rehabilitation Center of the Pensionsversicherungsanstalt in St. Radegund. The standard period of rehabilitation in St. Radegund is four weeks. In this study, the patients were randomly assigned to one of the three groups using a free, demo version of online software https://www.randomizer.at (accessed on 30 September 2019) for block randomization. This study was approved by the ethics committee and preregistered as a pilot clinical study at clinicaltrials.gov. The clinical study and data collection were performed in accordance with the Declaration of Helsinki (2013) of the World Medical Association. Every patient received detailed information about the study protocol and provided verbal and written informed consent. The flow of the study participants is shown in Figure 1.

### 2.1. Participants

Over the duration of two weeks, patients who arrived at the clinic to start their 4-week rehabilitation program and who met the strict inclusion criteria were enrolled in this study (Figure 1). On the day of admission, all patients underwent a clinical examination (general medical history and clinical examination), including ECG. The medications which were taken by the patients in the study included cardiovascular agents (such as ACE inhibitors, beta blockers, calcium channel blockers, diuretics, and statins), analgesics (such as paracetamol, ibuprofen, and diclofenac), antiplatelet and anticoagulant agents (such as aspirin, clopidogrel, and warfarin), respiratory agents (such as bronchodilators and corticosteroids), and gastrointestinal agents (such as proton pump inhibitors, H2 blockers, and antacids).

**Figure 2:**
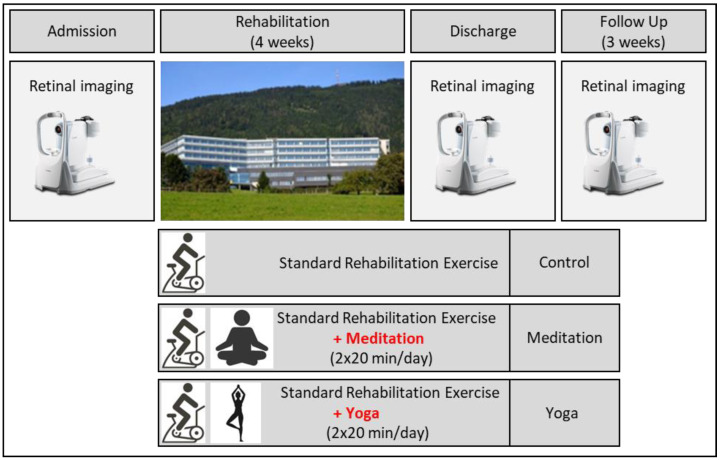
Overview of the study protocol (adapted from Rudlof and colleagues [21]).

This study employed a parallel-group randomized controlled trial design with an allocation ratio of 1:1:1. The thirty patients included were randomly assigned to three study groups—control, meditation, and yoga (Figure 2, Table 1). While the control group only received the standard rehabilitation exercise program, TM and yoga groups received, in addition to the standard rehabilitation therapy, either double the amount of meditation or yoga sessions of 20 min per day, respectively (Figure 2). The given groups performed meditation or yoga exercises according to a plan, as mentioned later and in the work of Rudlof and colleagues [21].

### 2.2. Sample Size

As there were no other studies we were aware of that investigated the effect of TM and/or yoga in the setting of cardiac rehabilitation, we conducted a pilot study with 30 participants (10 in each group). The primary outcomes measured included changes in endothelial function, which were assessed through various modalities such as flow-mediated dilatation, pulse wave velocity, retinal imaging, and cardiopostural interactions.

### 2.3. Eligibility Criteria

Participants had to meet the following criteria to be included in this study. They needed to be recovering from one of these conditions: myocardial infarction, ST-elevation myocardial infarction, non-ST-elevation myocardial infarction, acute coronary syndrome, coronary artery disease with percutaneous coronary intervention, or a coronary artery bypass graft. The inclusion age was from 40 to 80 years, and participants had to be inpatient admissions at the Cardiac Rehabilitation Center St. Radegund. The exclusion criteria were as follows: patients who had to be monitored because of severe disease; patients with NYHA III or more; patients with a mini-mental score of less than 26; patients who were not sufficiently mobilized; and patients who regularly perform any kind of meditation techniques. No study participants suffered from retinopathies or eye problems.

### 2.4. Standard Rehabilitation Exercise Therapy

During the 4 weeks of cardiac rehabilitation, the patients received an individual therapy plan. The individual rehabilitation schedule was created for each patient according to the results of health check tests and their fitness level/condition/performance. The individual performance was assessed via cyclic ergometry. The rehabilitation program was multidisciplinary, containing an exercise part, physiotherapeutic treatment, nutrition support, psychological assistance, and rehabilitation nursing. The following exercises were available for the patients: bicycle or treadmill (with ECG monitoring); Nordic walking, hiking (in groups); gymnastics; strength, endurance, and mobility workouts; weightlifting under physiotherapeutic control; and swimming. All therapeutic units had a duration of 25 or 50 min. The exercise modality followed a standardized procedure. In general, the patients underwent 2400 min of standard rehabilitation exercise therapy in 4 weeks. All diagnostic and therapeutic processes were performed following the latest guidelines of the European Society of Cardiology (ESC).

The physiotherapeutic individual treatment included, for example, massages, lymphatic drainages, electro-therapy, etc. Nutritional support included education about mixed healthy diets provided by dieticians, including various educational seminars (such as lectures concerning cardiovascular risk factors). Psychological assistance, employing stress-reducing methods, was free to access for all patients depending on their individual needs. Additionally, relaxation sessions that patients could attend comprised progressive muscle relaxation, autogenic training, acupuncture massage, and dry water massage. The relaxation schedule was individually adapted depending on the patient’s condition and preferences.

The staff members were trained for this special group of patients. Medical doctors, mainly specialists in internal medicine/cardiology or general practitioners, therapeutic staff, psychologists, dietologists, and nurses gave support in multiple aspects of patients’ lives. The object of the therapeutic and diagnostic procedures is the restoration and strengthening of workability and participation in everyday life.

### 2.5. Standard Rehabilitation Exercise Therapy + Transcendental Meditation

In the beginning, before the study started, the patients received four introductory transcendental meditation lectures in which the method was learned via practice. Two TM professionals of the “Austrian Society of Maharishi Vedic Sciences” (Österreichische Gesellschaft für Maharishi Vedische Wissenschaft) held these introductory transcendental meditation lectures in which the participants learned the technique. Each of these first four introductory sessions lasted for about 1–1.5 h and took place on 4 consecutive days. To correctly learn the method, it was necessary to complete all four sessions in a row.

After the introductory lectures, the TM technique was practiced twice a day for 20 min, with one session in the morning (6:30–6:50) and one in the evening (16:30–16:50). Two group meetings per week and one individual personal meeting with the TM teacher (this allowed the patients to ask questions) guaranteed the right performance of TM by the patients.

### 2.6. Standard Rehabilitation Exercise Therapy + Yoga

In the beginning, before the study started, the patients in this group received a 20 min workshop where they learned how to perform yoga exercises. The yoga workshop was held by a yoga expert.

Afterward, yoga sessions were included in the regular/standard exercise. The yoga group performed yoga, which included physical poses (Asanas), body scan, breathing exercises (Pranayamas), and affirmations, at the same time slots as those described for the TM group. It was practiced twice a day for 20 min, with one session in the morning (6:30–6:50) and one in the evening (16:30–16:50).

### 2.7. Study Protocol

Patients were tested at three separate time points: the first measurement (admission/baseline measurement) was taken one day after admission and before any kind of rehabilitation, the second measurement was taken four weeks later (discharge measurement) at the end of the rehabilitation program, and the third measurement (follow up measurement) was conducted three weeks after discharge (Figure 2). The measurements were performed within one day of each other at fixed times (9:00, 10:30, 13:00, 14:30, and 16:00) and were individually repeated at the same time (admission, discharge, and follow-up interventions/control). While the current study involves the same number of participants as the large comprehensive project, it serves as a pilot study in terms of measurement comprehensiveness. The present pilot study focused solely on the retinal microcirculation approach, providing a unique perspective within the broader scope of the main project. The whole project encompasses a broader range of measurements, for example, tension myography [48] and cardiovascular parameter evaluation using the Task Force Monitor^®^ (CNSystems, Graz, Austria) [21]. The detailed protocol is shown in the previous research [21].

### 2.8. Retinal Assessment

**Figure 3:**
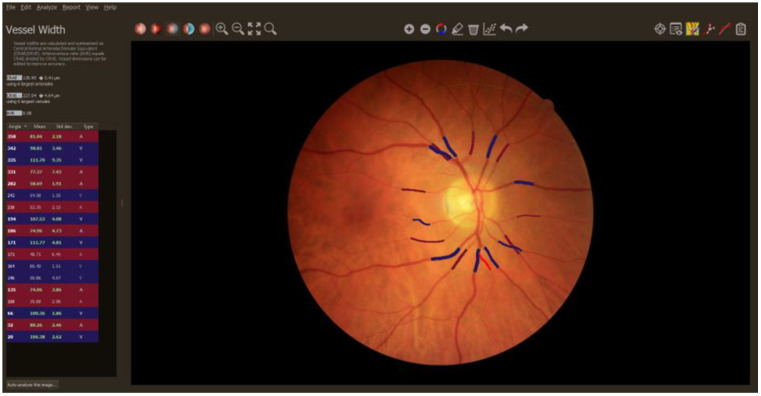
An optic disc focused image of the retina displayed in software (version 2.1.1) MONA-REVA during the analysis process. The table on the left side of the picture shows the list of the analyzed vessels. Arteries are displayed in red and venules are shown in blue. Boldly displayed vessels in the table, which correspond to the six biggest arteries and six largest venules from the picture, were used for the calculation of CRAE and CRVE. Central retinal artery equivalent (CRAE); central retinal vein equivalent (CRVE).

The retinal images (resolution of 1536 × 1536) focused on the optic disc of the right eye were collected at 3 points according to the study protocol. They were taken by a trained person using a non-mydriatic digital retinal camera, Canon CR-2 (Canon Medical Systems Europe B.V., Amstelveen, The Netherlands). This was followed by the allocation, organization, and preparation of retinal images for analysis. The analysis was performed by a trained grader blinded from any previous knowledge about the details of the study using the semi-automated software (version 2.1.1) MONA Reva (VITO, Boeretang, Mol, Belgium). The diameters of retinal microvessels in an area 0.5 to 1 the size of the optic disc radius, determined from the optic disc margin, were automatically analyzed. Subsequently, the trained grader checked, corrected, and labeled vessels (arterioles, venules). The Knudson formula was used to calculate retinal parameters ((central retinal artery equivalent (CRAE), central retinal vein equivalent (CRVE) and artery-to-vein ratio (AVR)) from the 6 largest retinal arterioles and the 6 largest retinal venules [49,50,51,52,53,54,55]. An example of the fundus image taken during the study is displayed in Figure 3.

### 2.9. Statistical Analysis

All retinal data were checked to determine the normality of the distribution via the Shapiro–Wilk test. For the comparison of retinal parameters at the three different time points, repeated measures ANOVA was performed, using the rehabilitation type as the between-subject factor. Statistical significance was determined in relation to a two-tailed *p*-value of *p* < 0.05. Data were analyzed using IBM SPSS Version 28.0 (IBM Corp., Armonk, NY, USA). Continuous variables are expressed as mean ± standard deviation (SD).

## 3. Results

Overall, 30 patients (24 males, 6 females; mean age: 59.36 (±9.22); height: 173.34 (±8.12); weight: 84.59 (±20.23); and BMI: 27.99 (±5.59)) were recruited in this pilot, randomized clinical study (Table 1). In total, 2 patients dropped out at the beginning of the study (without further explanation), and 13 additional dropouts (caused by low quality of retinal images) were noticed during follow-up measurements. Therefore, the follow-up measurements could be only performed with 50% of the patients (5 in each group). Table 1 summarizes the basic characteristics of the study participants.

### Retinal Microvasculature

Table 2 summarizes the analyzed retinal parameters of the three groups.

Considering CRAE, the repeated measures ANOVA revealed neither a main effect for time point (admission, discharge, follow-up: F(2,24) = 0.890, *p* = 0.424) nor a group effect (F(2,12) = 1.236, *p* = 0.325), and the interaction was also non-significant (F(4,24) = 0.600, *p* = 0.666). Similarly, we found no main effect for the time point (F(2,22) = 0.483, *p* = 0.620), group (F(2,11) = 0.500, *p* = 0.620) or the interaction (F(4,22) = 1.477, *p* = 0.243) for CRVE. Also, the analysis of the AV ratio did not show statistical significance for any effect (time point: F(2,22) = 1.821, *p* = 0.185; group: F(2,11) = 0.788, *p* = 0.479; interaction: F(4,22) = 1.316, *p* = 0.295). Overall, we saw no significant effect for intervention, and thus rehabilitation, or between patients’ groups with regard to retinal microcirculation parameters.

Due to the large drop out of patients in the follow-up measurements, we also only compared the time points—admission and discharge—that were available for 28 patients. However, the statistical analyses correspondingly showed no significant differences between the three groups (Table 3).

For CRAE measurements, there were no significant effects for time point (pre-post: F(1,25) = 0.001, *p* = 0.970), group (F(2,25) = 0.439, *p* = 0.649), or their interaction (F(2,25) = 0.066, *p* = 0.936). Similarly, and also for the CRVE measurements, no significant changes were observed between admission and discharge (F(1,25) = 1.794, *p* = 0.193), group (F(2,25) = 0.146, *p* = 0.865), or their interaction (F(2,25) = 0.106, *p* = 0.900). Also, the AV ratio showed that the factors included had no effect (admission–discharge: F(1,25) = 1.891, *p* = 0.181; group: F(2,25) = 0.143, *p* = 0.867; interaction: F(2,25) = 0.028, *p* = 0.972).

## 4. Discussion

We did not find any differences in retinal microcirculation parameters over time between the three rehabilitation groups: those receiving standard rehabilitation exercise program only, those practicing yoga, and those practicing TM. Nonetheless, our results are novel and encourage future research as there is an evident lack of peer-reviewed articles from the Western world covering topics of meditation and yoga and no previous study has examined cardiovascular health via retinal imaging—a promising, non-invasive, and cost-effective technique [42]—in cardiac patients during cardiac rehabilitation.

The positive effects of exercise on cardiovascular health are very well known [56,57,58,59,60,61]. The acute effects of exercise are manifested via the elevation in cardiac stroke volume and HR, an increase in cardiac output, which coupled with a transient increase in systemic vascular resistance, increases mean arterial BP. However, it was confirmed by numerous quantitative studies that long-term exercise not only promotes a reduction in HR and BP at rest but also has an impact on a lot of other parameters related to cardiovascular health. Moreover, different types of exercise approaches are important therapeutic treatments for patients with cardiovascular diseases and help in rehabilitation after cardiac surgeries. Hambrecht and colleagues investigated the effect of exercise on coronary endothelial function in 19 CAD patients randomized to either an exercise-training group (10 patients) or a control group (9 patients) [58]. After 4 weeks of study, increased response to acetylcholine and a 29% increase in coronary blood-flow reserve were observed in the exercise-training group, which showed improvement in endothelium-dependent vasodilatation. A meta-analysis from Fagard showed that dynamic physical training, performed 3–5 times (30–60 min/session) per week, decreases BP by an average of 3.4/2.4 mmHg [59]. A systematic review and meta-analysis of 48 trials (6 or more months of follow-up) with 8940 patients concluded that, compared with usual care, exercise-based cardiac rehabilitation is associated with reduced all-cause mortality, better improvement in lipid profile, a reduction in systolic BP (SBP), and lower rates of self-reported smoking [60]. Another meta-analysis involved 72 trials, 105 study groups, and 3936 participants and investigated the effects of endurance training on BP [61]. It was found that training reduces resting and daytime ambulatory BP by 3.0/2.4 mm Hg and 3.3/3.5 mm Hg, respectively. Moreover, the reduction in resting BP was even higher in the hypertensive study groups (−6.9/−4.9 mm Hg). Other results were seen in the reduction in systemic vascular resistance by 7.1%, plasma norepinephrine by 29%, and plasma renin activity by 20%. Body weight dropped by 1.2 kg, waist circumference fell by 2.8 cm, percent body fat fell by 1.4%, and the homeostasis model assessment index of insulin resistance fell by 0.31 IU/L^−1^. Additionally, HDL increased by 0.032 mmol/L^−1^. With regard to these results mentioned above, the effects of aerobic endurance training on cardiovascular health, via a favorable impact on cardiovascular risk factors, are evident. It is possible that similar effects were seen in our study cohorts, as all three groups that we studied received similar but rigorous standard exercise therapy. However, we did not observe any significant changes in retinal microcirculation parameters, not even between three different time points. Moreover, the retinal imaging technique is a sensitive approach, especially when it comes to sample size. On the other hand, individualized and non-uniform exercise training, which has a lot of different exercise options to choose from, could be also an important factor that limits the capture of significant results.

In a systematic review and meta-analysis, Chu and colleagues looked at the effectiveness of yoga in modifying risk factors for cardiovascular disease, and a no-exercise group and an exercise group were used as controls [22]. When they compared the yoga group with non-exercise controls, the yoga group showed significant improvement in BMI (−0.77 kg/m^2^), SBP (−5.21 mmHg), LDL (−12.14 mg/dL), and HDL (3.20 mg/dL), as well as in body weight (−2.32 kg), diastolic BP (DBP) (−4.98 mmHg), total cholesterol (−18.48 mg/dL), triglycerides (−25.89 mg/dL), and HR (−5.27 beats/min). However, there was no significant difference between the yoga and exercise approaches. Therefore, yoga seemed to be a comparably effective treatment for improving cardiovascular profile via exercise. Also, Murugesan and colleagues reported that yoga has a positive effect on the cardiovascular system and is effective in the treatment of hypertension [62]. Thirty-three hypertensives (35–65 years) participated in a clinical trial and were assigned to three groups (1. yoga exercise, 2. medical treatment, 3. neither yoga nor medical treatment). The yoga group performed yoga exercises for one hour in the morning and in the evening, and the medical treatment group took one drug every day for 11 weeks. The capability to control hypertension was investigated for both groups. Both treatments, yoga as well as medications, were effective in controlling hypertension as the study participants showed significant decreases in SBP, DBP, pulse rate, and body weight. Our study participants took part in yoga exercises twice a day for 20 min over the course of 4 weeks. It is possible that the shorter daily time of yoga practice as well as the total duration of the study, only 4 weeks compared to 11 weeks in the study mentioned above, prevented us from seeing any significant effects in the parameters of retinal microcirculation. Moreover, even though yoga has an impact on psychological well-being, stress reduction, and overall quality of life, we did not measure those aspects. This could, potentially pose confounders or variables that were not controlled for in the study. Unaccounted psychological factors, subjective well-being, and heterogeneity in participant responses to yoga may also partially explain our non-significant results.

A systematic review and meta-analysis investigating the impact of yoga on cardiovascular disease risk factors showed the beneficial effects of yoga (*p* < 0.05) on SBP (reduced by 5.85 mm Hg), DBP (reduced by 4.12 mm Hg), HR (reduced by 6.59 beats/min), respiratory rate (reduced by 0.93 breaths/min), abdominal obesity (decrease in waist/hip ratio and waist circumference), lipid profile (decrease in LDL, total cholesterol, triglycerides and increase in HDL), hemoglobin A1c (reduced by 0.45%),O and insulin resistance (reduced by 0.19) relative to usual care or no intervention [23]. Pal and colleagues investigated the effect of yoga practice on cardiovascular health in patients with coronary artery disease [63]. Overall, 170 patients were assigned to either a yoga group [35–40 min/day, five days/week) or a non-yoga group for six months. They also noted significant reductions in BMI, SBP, DBP, HR, total cholesterol, triglycerides, and LDL in the yoga group. Therefore, yoga may be a helpful intervention for patients with coronary artery disease. Despite an identical number of yoga sessions, the total duration of the study was six months, and was hence six times longer than the present study. This may be the reason we did not achieve significant changes in the variables we investigated.

Another study included 130 patients with heart failure and assigned them to a yoga group (standard medical therapy + yoga (60 min/day, three days/week, a total of 36 sessions)) or a control group (standard medical therapy) [27]. They observed a significant reduction in HR and BP in the yoga group compared to the control group. Moreover, the reduction in LF and LF-HF ratio and elevation in HF in the yoga group showed improved parasympathetic activity and decreased sympathetic activity. Although this study took 12 weeks, the overall duration of the yoga exercises during the whole study was similar to ours. Compared to the present study, yoga exercises were more intense for our patients; therefore, we would expect to see significant changes. However, while their study included 130 participants, our pilot study had only 30, with additional dropouts recorded during the protocol. Those and the already abovementioned reasons may be behind our non-significant results.

A case-control study included 100 subjects (50 controls (not doing any type of physical exercise), and 50 study subjects (practicing yoga for 5 years)) above 40 years of age [64]. They found that the study group had significantly lower SBP and DBP. Five years and one month is a very big difference in study duration, and this is probably the reason why similar results were not reflected in our measurements. Slower breathing rate is a typical part of yoga exercises. A total of 79 mild hypertensive individuals with BP > 140/90 mm Hg were enrolled in a study and assigned to either the treatment or control group [65]. Patients in the treatment group performed a 15 min/day session of device-guided breathing exercises. We obtained BP and HR results, self-measured at home every morning throughout the 10-week, from both groups. Device-guided breathing exercises caused a reduction in SPB and DBP (5.5/3.6 mm Hg at the clinic and 5.4/3.2 mm Hg at home). Although this study did not deal directly with yoga exercises, the slower breathing rate is a typical part of yoga exercises. The daily duration was shorter, but the study lasted 2.5 times longer compared to the present study. Perhaps a longer period of time with shorter daily duration of breathing exercises, compared to a shorter intervention period and more intense exposure to yoga, has a more beneficial effect.

The beneficial effects of TM have previously been published [66]. Dillbeck and Orme-Johnson observed acute reductions in respiratory rate and plasma lactate in the TM group compared to the control group, which was characterized by increased basal skin resistance. Moreover, we observed lower baseline HR, respiratory rate, plasma lactate level, and spontaneous skin resistance responses outside of meditation in the TM group. Mindfulness-based stress reduction (MBSR) is an eight-week program offering intensive mindfulness training to assist people with stress, anxiety, or depression. Fifty-six patients with unmedicated prehypertension were randomly assigned to MBSR or progressive muscle relaxation (PMR) groups [17]. The study lasted for 8 weeks, with treatment sessions of 2.5 h each week. The MBSR group showed a 4.8 mm Hg and 1.9 mm Hg reductions in SBP and DBP, respectively, compared to a 0.7 mm Hg reduction in SBP and 1.2 mm Hg increase in DBP in the PMR group. The total duration of meditation during the study was comparable to the present study, but our study lasted half as long (4 weeks vs. 8 weeks) and the exposure to meditation was therefore less intense. Similar to what we saw with yoga, perhaps a longer and less intensive form of therapy may have a greater effect. Moreover, similarly, as was mentioned in the discussion of yoga, the absence of measurements related to psychological well-being, stress reduction, and overall quality of life in the study may potentially present confounding issues that were not controlled for in the analysis of retinal microcirculation.

A single-blind study with a 3 month follow-up investigated the effect of 3 different approaches (TM, PMR, or lifestyle modification exercise) on the treatment of mild hypertension in 127 elderly African Americans (aged 55 to 85 years) [67]. It was observed that both TM and PMR reduced SBP (10.7 mmHg and 4.7 mmHg) and DBP (6.4 mmHg, 3.3 mmHg). However, the TM approach was approximately twice as effective when the reductions in the TM group were significantly greater than those seen in the PMR group. As in present study, the subjects conducted TM twice daily (morning and evening) for 20 min per session. The differences lie in the length of the Schneider study (3 months [67] vs. our intervention duration of only 4 weeks). This, along with multiple reasons already mentioned, could have contributed to the lack of changes in retinal microcirculation parameters in our patient population.

A meta-analysis from 2008 including normotensive, prehypertensive, and hypertensive individuals (ages from adolescent to senior) compared BP changes in TM and the control group [25]. Average reductions of 4.7 mmHg in SBP and 3.2 mmHg in DBP in TM groups were observed when compared with control groups. Additionally, subgroup analyses of hypertensive groups and high-quality studies showed similar BP values. Another meta-analysis performed by Rainforth and colleagues investigated the effects of stress reduction programs (biofeedback, relaxation-assisted biofeedback, progressive muscle relaxation, stress management training, and TM program] on changes in BP [68]. To sum up, only TM showed a significant reduction in SBP (5 mmHg, *p* = 0.002) and DBP (2.8 mmHg, *p* = 0.02). Two additional studies showed that TM also has beneficial effects on insulin resistance and lipid profiles. One of the studies lasted for 16 weeks, with 103 participants with coronary heart disease (CHD), and was randomized to either TM or a health education (HE) group to examine the efficacy of TM on components of metabolic syndrome and CHD [18]. The TM group had two introductory lectures (1.5 h each), personal interview (10–15 min), personal instruction (1–1.5 h), 3 group meetings (1.5 h each), and follow-up and maintenance meetings (1.5 h) twice per week during the first 4 weeks and then weekly. The HE group had the same number, length, and frequency of group meetings with health educators. The TM group showed favorable changes in SBP (*p* = 0.04), insulin resistance (*p* = 0.01), and HRV (HF; *p* = 0.07) compared with the control group. The increasing HF values suggests that there is autonomic regulation, particular increased parasympathetic activity, involved in the mechanism of TM’s effect on cardiovascular health. Additionally, a small study where Cooper and Aygen compared 12 patients with hypercholesterolemia regularly practicing TM (20 min/day, 13 months) with 11 control subjects displayed a 10% (*p* < 0.005) reduction in fasting serum cholesterol levels in the TM group compared with the control group [24]. The first of these two studies again had a longer duration, as well as less frequent sessions and a bigger sample size, than our own. All these factors could have contributed to the differences from the findings in our study. The second study was like the present study only with a small sample size. However, they had shorter meditation sessions per day (20 min vs. 2 × 20 min in present study). However, the overall duration of the respective study’s meditation therapy was 13 months, which is a big difference compared to our 4-week intervention. It cannot be forgotten that the retinal imaging technique is a sensitive approach, especially when it comes to sample size, and together with the mentioned confounders and other reasons may contribute to the non-significant results of the present study.

Previous studies have also shown the effect of these two alternative approaches on HRV and the autonomic nervous system. Yoga, as well as TM, showed a boost in parasympathetic activity and the silencing of sympathetic activity, which is considered the main their mechanism of action affecting the cardiovascular system [12,24,25,26,27]. We did not, however, measure autonomic function via heart rate variability.

## 5. Summary

To summarize the results of the present study, we did not observe significant changes in retinal microvasculature as an effect of three different cardiac rehabilitation approaches, neither between rehabilitation groups nor between different measurement time points. Previous research proved that exercise has a favorable impact on cardiovascular risk factors. It improves the ability of the mitochondrion to adjust cardiac metabolism, causes a reduction in oxidative stress, and increases the cardiac reserve capacity. Based on the previous research, we hypothesized that four weeks of cardiac rehabilitation program would be enough to transform cardiovascular changes into changes in retinal microvasculature. Moreover, we compared the standard exercise rehabilitation approach with either yoga or TM, and also compared these approaches with each other. Similarly, as an exercise, the positive effects of meditation and yoga on mental as well as cardiovascular health are known [9,10,11,12,19,20,21]. We believed that four weeks of yoga or meditation in addition to standard rehabilitation would be strong enough to surpass the effect of standard exercise rehabilitation itself. We expected these cardiovascular changes to be transformed into changes in retinal microvasculature. This novel approach to measuring the physiological effects in cardiac patients undergoing cardiac rehabilitation and retinal imaging presents a promising, non-invasive, and cost-effective technique for cardiovascular health evaluation [28,29,30,31,32,33,34,35,36,37].

We believe that the small sample size, with additional dropouts in follow-up measurements as well as differences in the duration of sessions and overall interventions, could have masked the significant results that were expected. According to the previous research, it was observed that perhaps a longer and less intensive therapy may have a greater effect. Furthermore, individualized and non-uniform exercise training, with a lot of different exercise options to choose, from could be also an important factor that limits the capture of significant results. In addition, even though yoga and meditation have an impact on psychological well-being, stress reduction, and the overall quality of life, we did not measure these factors, and they can potentially present confounders or variables that were not controlled for in the study. The psychological factors unaccounted for, subjective well-being, and heterogeneity in participant responses to yoga and meditation may also partially explain our non-significant results. Finally, the retinal imaging technique is a sensitive approach, especially when it comes to sample size, and may contribute to difficulties with observing the significances within the present study.

## 6. Limitations

One of the limitations of the study is that we did not include a large number of patients. We believe that the small sample size could have masked significant results when three measurement time points were used, with only five patients per group. However, we do not think this is a problem, as the present study was a pilot study that aimed to assess microvascular changes. The obtained data will provide us with information (such as determining how possible the design of a study is in reality or allowing us to calculate sample sizes) for larger epidemiological studies. The medications that have been taken by the participants could be considered as a limitation that could affect the results of the study; however, cardiac patients typically take these types of medications. The present study groups were balanced in terms medication usage, keeping the quality of our study groups stable, and we do not believe that the medications taken by the patients in the present study affected the results of the study. Moreover, in this repeat measurement study, the same patients were assessed pre- and post-intervention. Another limitation of the present clinical study is that we could not carry out the follow-up in all the patients. This was largely because the COVID-19 pandemic occurred during that period, which limited our access to patients, and hence we experienced a lot of missing data. A potential limitation of our study is also that the interventions (yoga and TM) were only applied for a brief period. A comparison of the present study results with other studies indicates that a longer and less intensive therapy (yoga/TM) could have a greater beneficial effect on retinal microvasculature than a shorter and more intensive approach. In addition, even though yoga and meditation have an impact on psychological well-being, stress reduction, and overall quality of life, we did not measure those aspects, and these factors can potentially present confounders or variables that were not controlled for in the study. Furthermore, individualized and non-uniform exercise training, with a lot of different exercise options to choose, from could be also an important factor and limit the capture of significant results. Lastly, the high sensitivity of the retinal imaging technique, especially when it comes to sample size, could also pose a limitation of this study as the method may not be able to capture differences in given parameters. However, we do not believe this was the case as all pictures were captured and analyzed by trained personnel of the Medical University of Graz.

Despite the above limitations, the present clinical study provides novelties in terms of rehabilitation approaches after cardiac surgery. In addition to the exercise protocols that are widely used during cardiac rehabilitation, non-pharmacological approaches, such as yoga and meditation, can also be incorporated. Furthermore, the present study presents novel results, as there is a lack of research in this area, and shows that cardiovascular health can be routinely assessed non-invasively by the retinal imaging approach. The combination of these underestimated rehabilitation approaches and the assessment by retinal imaging, which presents novelty and a promising approach in cardiovascular health evaluation, ensures the significance of the present pilot study. As our results were not conclusive, we suggest that future research should include longer periods of breathing exercises and yoga to examine the potential effects of yoga and TM on retinal microcirculation and overall cardiovascular health.

## 7. Conclusions

The present pilot study introduced complementary rehabilitation approaches, including yoga and meditation, for cardiac surgery patients, and explored the use of retinal imaging as a non-invasive method to evaluate cardiovascular health. Although no significant changes in retinal microcirculation were observed, the findings provide foundational insights for future studies examining the integration of health promotion practices such as yoga and meditation into cardiac rehabilitation. The study also highlights the need for more comprehensive research in this area, particularly from Western populations, and underscores the potential of retinal imaging techniques in assessing cardiovascular health. Furthermore, we encourage the research community to conduct larger-scale studies to validate these approaches. Studies should utilize longer intervention durations and more robust methodologies in order to better understand the physiological impacts of yoga and meditation on retinal vascular health.

## Figures and Tables

**Table 1:** Characteristics of the study sample, excluding two dropouts from the beginning of the study. Data are shown as mean ± SD.

Characteristics	Control	Meditation	Yoga	Total Sample
Male (n)	8 (89%)	8 (89%)	6 (60%)	22 (79%)
Female (n)	1 (11%)	1 (11%)	4 (40%)	6 (21%)
Age (years)	59.33 (±7.81)	59.56 (±7.84)	59.20 (±12.09)	59.36 (±9.22)
Height (cm)	176.72 (±7.11)	173.44 (±7.73)	170.20 (±8.79)	173.34 (±8.12)
Weight (kg)	94.73 (±9.95)	79.56 (±19.22)	80.00 (±25.00)	84.59 (±20.23)
BMI (kg/m^2^)	30.42 (±4.24)	26.31 (±5.10)	27.30 (±6.74)	27.99 (±5.59)

**Table 2:** The effect of meditation or yoga in addition to a standard rehabilitation exercise program on retinal arteriolar and venular diameters in patients after heart surgery. Data are shown as mean ± SD (control, meditation, Yoga) at the respective timepoints (admission, discharge, follow-up). N—number of patients in each group; Medit.—meditation; CRAE—central retinal artery equivalent; CRVE—central retinal vein equivalent; AVR—artery-to-vein ratio.

Parameter	Group	Admission	Discharge	Follow-Up	N	*p*-Value
CRAE (µm)	Control	131.30 ± 13.20	132.03 ± 16.48	133.75 ± 14.32	5	0.666
	Medit.	140.23 ± 8.74	139.11 ± 7.88	140.20 ± 8.86	5	
	Yoga	146.98 ± 14.66	142.75 ± 16.99	145.00 ± 12.02	5	
CRVE (µm)	Control	211.67 ± 12.30	205.21 ± 7.07	204.62 ± 5.40	5	0.243
	Medit.	213.70 ± 23.27	215.30 ± 20.30	217.01 ± 19.61	5	
	Yoga	210.49 ± 9.21	219.51 ± 17.11	215.56 ± 19.17	5	
AVR	Control	0.63 ± 0.08	0.63 ± 0.07	0.63 ± 0.07	5	0.295
	Medit.	0.67 ± 0.04	0.66 ± 0.04	0.66 ±0.01	5	
	Yoga	0.70 ± 0.05	0.65 ± 0.04	0.65 ± 0.05	5	

**Table 3:** The effect of meditation or yoga in addition to a standard rehabilitation exercise program on retinal arteriolar and venular diameters in patients after heart surgery. Data show group ± SDs (Control, Meditation, Yoga) at the respective timepoints (Admission, Discharge). Data are shown as mean ± SD. N—number of patients in each group; Medit.—Meditation; CRAE—central retinal artery equivalent; CRVE—central retinal vein equivalent; AVR—artery-to-vein ratio.

Parameter	Group	Admission	Discharge	N	*p*-Value
CRAE (µm)	Control	141.4 ± 15.78	141.91 ± 17.65	9	0.936
	Medit.	135.84 ± 12.23	135.84 ± 9.35	9	
	Yoga	138.56 ± 13.06	138.16 ± 11.38	10	
CRVE (µm)	Control	212.28 ± 8.41	215.56 ± 11.13	9	0.193
	Medit.	208.55 ± 24.45	212.23 ± 20.84	9	
	Yoga	212.82 ± 14.09	214.31 ± 13.98	10	
AVR	Control	0.67 ± 0.08	0.66 ± 0.06	9	0.181
	Medit.	0.66 ± 0.07	0.64 ± 0.06	9	
	Yoga	0.65 ± 0.06	0.65 ± 0.05	10	

## Data Availability

All data generated or analyzed during this study are included in this published article. For any specific additional inquiries or requests for further information, please contact the corresponding author of this manuscript.
